# NANO-LM: An updated scorecard for the clinical assessment of patients with leptomeningeal metastases

**DOI:** 10.1093/neuonc/noae171

**Published:** 2024-08-29

**Authors:** Emilie Le Rhun, Lakshmi Nayak, Mary Jane Lim-Fat, Roberta Rudà, Elena Pentsova, Peter Forsyth, Barbara J O’Brien, Matthias Preusser, Priya Kumthekar, Dieta Brandsma, Michael Weller

**Affiliations:** Departments of Neurology and Neurosurgery, Clinical Neuroscience Center, University Hospital and University of Zurich, Zurich, Switzerland; Center for Neuro-Oncology, Dana-Farber Cancer Institute, Boston, Massachusetts, USA; Division of Neurology, Sunnybrook Health Sciences Centre, University of Toronto, Toronto, Ontario, Canada; Division of Neuro-Oncology, Department of Neuroscience, University of Turin, Turin, Italy; Department of Neurology, Brain Tumor Service, Memorial Sloan Kettering Cancer Center, New York, New York, USA; Department of Neuro-Oncology, H. Lee Moffitt Cancer Center and Research Institute, Tampa, Florida, USA; Department of Neuro-Oncology, The University of Texas MD Anderson Cancer Center, Houston, Texas, USA; Division of Oncology, Department of Medicine 1, Medical University of Vienna, Vienna, Austria; The Lou and Jean Malnati Brain Tumor Institute, Robert H. Lurie Comprehensive Cancer Center of Northwestern University, Chicago, Illinois, USA; Department of Neuro-Oncology, Netherlands Cancer Institute, Amsterdam, The Netherlands; Department of Neurology, Clinical Neuroscience Center, University Hospital and University of Zurich, Zurich, Switzerland

**Keywords:** examination, NANO, neoplastic, neurological, response, scale

## Abstract

**Background:**

There are no validated tools for the clinical neurological assessment of patients with leptomeningeal metastases (LM). However, clinical examination during the course of the disease guides medical management and is part of response assessment in clinical trials. Because neuroimaging may not always be obtained owing to rapid clinical deterioration, clinical neurological assessment of LM is essential, and standardization is important to minimize rater disagreement.

**Methods:**

The Response Assessment in Neuro-oncology-LM group launched a 2-step process, aiming at improving and standardizing the clinical assessment of patients with LM. We report here on the first step the establishment of a consensus scorecard. The task force had 9 virtual meetings to define general recommendations on neurological assessment and selected domains of interest that should be tested.

**Results:**

Fourteen domains of neurological symptoms and signs were selected: level of consciousness, cognition, nausea and vomiting, vision, eye movement, facial strength, hearing, swallowing, speech, limb strength, limb ataxia, walking, and bladder bowel functions. For each item, a clear instruction on how to perform the assessment is provided with scoring criteria between 0 and 2. The general clinical status of the patient and use of steroids, pain medications, and antiemetics should be documented. Neurological sequelae from previous brain metastases or cancer treatment should be rated at the baseline evaluation; it should be specified when symptoms or signs may be related to a condition other than LM.

**Discussion:**

A revised Neurological Assessment in Neuro-Oncology-LM consensus scorecard for clinical assessment has been established. An international prospective validation study of the proposal is currently ongoing (NCT06417710).

**Key Points**

- A validated scorecard is currently missing.
- The RANO-LM group has agreed on an updated NANO-LM consensus scorecard.
- An international prospective validation study is currently ongoing.

**Importance of the Study**

Clinical assessment of patients with leptomeningeal metastases (LM) should be reliable and reproducible. A validated scorecard is currently missing. Patients may present with various symptoms and signs depending on the location of LM. A minimum list of domains should be explored at baseline and during the course of the disease. Clinical response is part of the overall LM response assessment. Clear instructions need to be provided to the examiner to obtain an adequate and reliable assessment when judging the efficacy of a therapeutic approach in LM, both in the context of clinical trials and in clinical practice. The RANO-LM group has agreed on an updated NANO-LM consensus scorecard. An international prospective study is currently ongoing to validate this updated scorecard.

Leptomeningeal metastases (LM) are usually a late and life-threatening complication in patients with metastatic cancer. They are estimated to occur in up to 10% of patients with metastatic cancer and are still probably underdiagnosed. The median survival is limited to a few months for the majority of patients. Several efforts to improve the diagnosis, management, and follow-up of patients with LM are ongoing.^1–3^ The number of trials dedicated to LM^4–10^ or brain metastasis trials including a study arm dedicated to patients with LM is also increasing.^11,12^

The clinical neurological assessment is key in disease monitoring, and standardization is essential because of the need for a reliable and reproducible local assessment. In contrast to imaging and pathological assessment, a central review of clinical evaluation is almost impossible unless these assessments are filmed for later assessment. Moreover, rapid clinical deterioration may be observed, making it sometimes difficult to obtain an MRI to objectively document progression. Therefore, clinical examination is key to evaluate LM during the entire course of the disease and, in some cases, the only tool to define the disease progression.

Both the Response Assessment in Neuro-oncology (RANO) group LM committee (LANO)^13^ and the European Association for Neuro-Oncology (EANO)- European Society for Medical Oncology (ESMO) guidelines^1^ recommend considering 3 key elements: (1) clinical presentation, (2) MRI presentation, and (3) cerebrospinal fluid (CSF) findings, for the response assessment in patients with LM. Both the RANO and the EANO ESMO groups recommend the use of standardized protocols and standardized scorecards. Heterogeneity in patient populations and imaging protocols, as well as lack of validation of assessment tools, may have contributed to the disappointing or inconclusive results of early randomized clinical trials performed more than 20 years ago.^14^ A validated MRI scorecard has now been developed between European Organisation for. Research and Treatment of Cancer (EORTC) and RANO^15^ and can be used in the setting of clinical trials.

A standardized response assessment would help to draw conclusions even in the context of negative trials to identify effective therapies for patients with LM and to inform future improved trial design. The RANO group is an academic international group of experts dedicated to the definition, validation, and improvement of criteria for response assessment, taking into account technological improvement and increased medical knowledge. The RANO-LM group aims at developing consensus and validated tools for response assessment in patients with LM.

Patients with LM may present with variable clinical symptoms and signs depending on the topography of LM. Typical symptoms and signs of LM include headache, nausea and vomiting, mental status changes, gait difficulties, cranial nerve palsies (eg, diplopia or visual disturbances; cranial nerves II, III, IV, and VI), hearing loss (cranial nerve VIII), radicular signs including weakness, voiding, and cauda equina problems, and focal or radiating (radicular) neck and back pain.^1^ Up to 5% of LM patients have a normal neurological evaluation.^16^

A clinical scorecard dedicated to the study of patients with LM had been proposed by the RANO-LM group but has never been validated.^13^ In parallel with the initial proposal of the LANO group, another RANO working group, the Neurological Assessment in Neuro-Oncology (NANO) group, proposed, the NANO scale, a standardized scorecard for the evaluation of patients with brain tumors in general^17^ (Supplementary Tables 1 and 2). The NANO scorecard includes 9 domains, whereas the first RANO-LM effort proposes to evaluate 11 domains. Six domains are shared between the NANO and RANO-LM scorecards: gait, strength, sensation, facial strength, level of consciousness, and behavior. In addition, the NANO scale includes the evaluation of ataxia, visual fields, and language, whereas the initial RANO-LM scorecard includes vision, eye movement, hearing, swallowing, or other neurological findings.^13^ These latter items may be more relevant in patients with LM than in patients with intraparenchymal lesions only.

The NANO scale went through a validation effort. The interobserver agreement for this scorecard showed a more than 90% interobserver agreement rate with kappa statistics ranging from 0.35 to 0.83 (fair to almost perfect agreement). However, considering the inclusion of specific neurological items in the neurological examination of patients with LM compared to patients with parenchymal brain tumors, it appears necessary to use a dedicated scorecard for patients with LM.^17^

Accordingly, the RANO-LM group planned to revise and prospectively validate a clinical scorecard adapted to patients with LM. The group opted to first revisit and revise the consensus of the initial RANO-LM scorecard, integrating experience made with the development of the NANO scale,^17^ and in a second step to validate the new proposal. Here the RANO-LM group presents the consensus obtained to revise the design of a scorecard for the neurological clinical assessment of patients with LM.

## Methods

The RANO-LM group launched a 2-step process, aiming at improving and standardizing the clinical assessment of patients with LM. The first step aimed at establishing a consensus scorecard. The proposed scorecard will be validated in a second step.

The objective of the first step was to assemble a set of clinical symptoms and signs to monitor the course of disease of patients with LM. The RANO-LM group had 9 virtual meetings to determine general recommendations on neurological assessment and to select domains of interest that should be tested.

General recommendations were first discussed to define consensus guidance on the essential clinical annotation needed to correctly assess the clinical neurological status and on the rating of neurological symptoms and signs at baseline that may already have been present before the diagnosis of LM and thus not or not exclusively related to LM.

Candidate domains were then identified. The proposals from the NANO scale^17^ and from the first RANO-LM proposal^13^ were reviewed. All candidate domains to assess the neurological status were discussed during the meetings. For each domain, the group discussed and decided on the clinical relevance for patients with LM.

Instructions and options for rating scores were then defined for selected items. Instructions for examiners on how to use the new NANO-LM scorecard were designed based on earlier instructions for the NANO score. Instructions and scores of other scales commonly used in neurology, such as the Expanded Disability Status Scale used for patients with multiple sclerosis,^18^ the Revised Amyotrophic Lateral Sclerosis Functional Rating Scale,^19^ the NIH Stroke Scale,^20^ the Health Utility Index classification system,^21^ and the modified Barthel activities of daily living,^22^ were also discussed.

General considerations, selected items, and instructions for rating were reviewed for each domain. Discrepancies were solved by discussion and consensus formation.

All available data are provided in the manuscript. No institutional review board or ethics committee approval was required for this manuscript.

## Results

### General Recommendations for the Neurological Assessment

Simple written instructions are presented on the scorecard to facilitate consistent assessment across raters (Table 1). The general status, Eastern Cooperative Oncology Group Performance Status (ECOG PS), or Karnofsky performance status should be documented. Medications such as corticosteroids, analgesics, and antiemetics may influence neurological symptoms and signs. Their use should thus be documented (yes/no/unknown). The dose of steroids in milligrams equivalent to dexamethasone on the day of the evaluation should be documented.

**Table 1.** NANO-LM scorecard

Date Patient ID Examinator	ECOG Performance Status Karnofsky Performance Status Consumption of steroids:	□ yes □ no □ unknown; if yes: dose in mg equivalent dexamethasone per day:			
	Consumption of pain killers:	□ yes □ no □ unknown			
	Consumption of antiemetics:	□ yes □ no □ unknown			
Item	Instructions		Scores	If abnormal	Evolution
Level of consciousness	Impaired level of consciousness should lead to the assessment of differential diagnoses of LM progression (eg, seizure). A complete neurological examination can be performed only in patients with normal level of consciousness. The clinical evaluation should be performed for scores 0 and 1, but should not be performed in a patient with a score of 2. In these latter situations, the feasibility of the clinical evaluation should be regularly reassessed during the windows allowed by the trial.		0 = Normal 1 = Drowsy, requires verbal stimulation 2 = Somnolent, requires tactile stimulation OR unarousable, coma □ not assessed □ not evaluable	□ related to LM □ possibly related to LM □ unrelated to LM If unrelated to LM, related to:	□ new onset □ significant PD □ unchanged □ resolved
Cognition	Mild to moderate: no interference with daily activity. Severe: need assistance in daily life because of cognitive disorders, significant impairment, no structured conversation, and not able to understand simple items during the visit in the absence of language disorders. Should be corrected at the end of the examination if needed. In case of loss of capacity to consent, a patient should stop the trial.		0 = Normal 1 = Mild to moderate cognitive impairment 2 = Severe cognitive impairment □ not assessed □ not evaluable	□ related to LM □ possibly related to LM □ unrelated to LM If unrelated to LM, related to:	□ new onset □ significant PD □ unchanged □ resolved
Nausea and vomiting			0 = Normal, no treatment 1 = Mild to moderate, able to eat or drink 2 severe, not able to eat or drink □ not assessed □ not evaluable	□ related to LM □ possibly related to LM □ unrelated to LM If unrelated to LM, related to:	□ new onset □ significant PD □ unchanged □ resolved
Vision	Patients who require corrective lenses should be evaluated wearing adapted corrective lenses. Right and left eyes should be tested separately, score should reflect worse-performing eye. Normal refers to normal reading with glasses or contact lenses used in everyday life.		Right (R) Left (L) 0= Normal 0= Normal 1= Mild to severe visual loss 1= Mild to severe visual loss 2= Blind (no light perception) 2= Blind (no light perception) □ not assessed □ not assessed □ not evaluable □ not evaluable	□ related to LM □ R / □ L □ possibly related to LM □ R / □ L □ unrelated to LM □ R / □ L If unrelated to LM, related to:	□ new onset □ R / □ L □ significant PD □ R / □ L □ unchanged □ R / □ L □ resolved □ R / □ L
Eye movement	Right and left eyes should be tested separately, score should reflect worse-performing eye. Severe impairment requests a constant double vision. Should reflect the impairment in daily life.		Right (R) Left (L) 0 = Normal 0 = Normal 1 = Mild to moderate impairment 1 = Mild to moderate impairment 2 = Severe impairment 2 = Severe impairment □ not assessed □ not assessed □ not evaluable □ not evaluable	□ related to LM. □ R / □ L □ possibly related to LM □ R / □ L □ unrelated to LM □ R / □ L If unrelated to LM, related to:	□ new onset □ R / □ L □ significant PD □ R / □ L □ unchanged □ R / □ L □ resolved □ R / □ L
Facial strength	Weakness includes nasolabial fold flattening, asymmetric smile, and difficulty elevating eyebrows. Right and left sides should be evaluated separately, score should reflect worse-performing side (last to be confirmed). Mild/moderate weakness means incomplete palsy. Severe facial weakness means complete palsy.		Right (R) Left (L) 0 = Normal 0 = Normal 1 = Mild to moderate weakness 1 = Mild to moderate weakness 2 = Severe facial weakness 2 = Severe facial weakness □ not assessed □ not assessed □ not evaluable □ not evaluable	□ related to LM □ R / □ L □ possibly related to LM □ R / □ L □ unrelated to LM □ R / □ L If unrelated to LM, related to:	□ new onset □ R / □ L □ significant PD □ R / □ L □ unchanged □ R / □ L □ resolved □ R / □ L
Hearing	Normal: able to hear and converse with evaluator easily without any difficulty Right and left ears should be tested separately, score should reflect worse-performing ear (last to be confirmed). Mild to moderate impairment can be related to a deficit in hearing or to tinnitus.		Right (R) Left (L) 0 = Normal 0 = Normal 1 = Mild to moderate impairment 1 = Mild to moderate impairment 2 = Unable to hear 2 = Unable to hear □ not assessed □ not assessed □ not evaluable □ not evaluable	□ related to LM □ R / □ L □ possibly related to LM □ R / □ L □ unrelated to LM □ R / □ L If unrelated to LM, related to:	□ new onset □ R / □ L □ significant PD □ R / □ L □ unchanged □ R / □ L □ resolved □ R / □ L
Swallowing	No bedside testing should be performed.		0 = Normal 1 = Occasional choking or modification of diet related to swallowing disorders 2 = Need tube feeding □ not assessed □ not evaluable	□ related to LM □ possibly related to LM □ unrelated to LM If unrelated to LM, related to:	□ newly onset □ significant PD □ unchanged □ resolved
Speech	Corresponds to the evaluation of articulation (speech) and should not be confused with a language impairment.		0 = Normal 1 = Mild to moderate, patient can be understood with difficulties 2 = Severe, patient is unintelligible □ not assessed □ not evaluable	□ related to LM □ possibly related to LM □ unrelated to LM If unrelated to LM, related to:	□ newly onset □ significant PD □ unchanged □ resolved
Strength	Test each limb separately: right arm/leg and left arm/leg side testing. It is recommended to assess proximal (above knee or elbow) and distal (below knee or elbow) major muscle groups. Score should reflect worst-performing area. Patients with baseline level-2 function in one major muscle group/limb should be scored on assessment of other muscle groups/limb.		Arm Right (R) Left (L) 0 = Normal 0 = Normal 1 = Mild to moderate weakness 1 = Mild to moderate weakness 2 = Severe weakness 2 = Severe weakness □ not assessed □ not assessed □ not evaluable □ not evaluable	□ related to LM □ R / □ L □ possibly related to LM □ R / □ L □ unrelated to LM □ R / □ L If unrelated to LM, related to:	□ new onset □ R / □ L □ significant PD □ R / □ L □ unchanged □ R / □ L □ resolved □ R / □ L
	Mild to moderate: Movement present but decreased against resistance. Severe: Movement present but none against resistance or no movement.		Leg Right (R) Left (L) 0 = Normal 0= Normal 1 = Mild to moderate weakness 1 = Mild to moderate weakness 2 = Severe weakness 2 = Severe weakness □ not assessed □ not assessed □ not evaluable □ not evaluable	□ related to LM □ R / □ L □ possibly related to LM □ R / □ L □ unrelated to LM □ R / □ L If unrelated to LM, related to:	□ new onset □ R / □ L □ significant PD □ R / □ L □ unchanged □ R / □ L □ resolved □ R / □ L
Limb ataxia	Nonevaluable if strength is too compromised (strength at 2). Trunk and lower extremities are assessed by Walking domain. Score based on the best response of at least 3 attempts. Normal: Able to finger-to-nose touch without difficulty. Mild to moderate: Able to finger-to-nose touch but difficult. Severe: Unable to finger-to-nose touch.		Right (R) Left (L) 0 = Normal 0 = Normal 1 = Mild to moderate difficulty 1 = Mild to moderate difficulty 2 = Severe difficulty 2 = Severe difficulty □ not assessed □ not assessed □ not evaluable □ not evaluable	□ related to LM □ R / □ L □ possibly related to LM □ R / □ L □ unrelated to LM □ R / □ L If unrelated to LM, related to:	□ new onset □ R / □ L □ significant PD □ R / □ L □ unchanged □ R / □ L □ resolved □ R / □ L
Walking	Walking is ideally assessed by at least 10 steps.		0 = Normal 1 = Abnormal but walks without assistance or abnormal and requires assistance (companions, cane, walker) 2 = Unable to walk □ not assessed □ not evaluable	□ related to LM □ possibly related to LM □ unrelated to LM If unrelated to LM, related to:	□ newly onset □ significant PD □ unchanged □ resolved
Cauda equina symptoms bowels			0 = Normal 1 = Occasional accident (max once a week) 2 = Incontinent or obstipation with a need for manual evacuation □ not assessed □ not evaluable	□ related to LM □ possibly related to LM □ unrelated to LM If unrelated to LM, related to:	□ newly onset □ significant PD □ unchanged □ resolved
Cauda equina symptoms bladder			0 = Normal 1 = Occasional accident (max once per 24 hours, pads) 2 = Incontinent, catheter needed □ not assessed □ not evaluable	□ related to LM □ possibly related to LM □ unrelated to LM If unrelated to LM, related to:	□ newly onset □ significant PD □ unchanged □ resolved
General overall assessment	Relative to the best overall clinical evaluation obtained during follow-up. CR: resolution of all signs and symptoms for all domains, only if the clinical evaluation was initially abnormal. SD: all other situations. PD 0 → 2: in any domain, or 0 1 OR 1 → 2: in at least 3 domains. Progression can be defined only if the neurological symptom or sign is related to LM. If worsening is considered as ‘possibly related to LM’, and then later confirmed as ‘related’: the date of PD should be backdated.		Relative to previous assessment □ CR □ PD □ SD Relative to baseline assessment □ CR □ PD □ SD	□ related to LM □ possibly related to LM □ unrelated to LM If unrelated to LM, related to:	

Abbreviations: CR = complete response; ECOG = Eastern Cooperative Oncology Group; LM = leptomeningeal metastases; PD = progressive disease; SD = stable disease.

The task force proposes that neurological sequelae from previous brain metastases or cancer-directed therapy, such as symptoms and signs from neuropathy or related to post-radiotherapy toxicities, should be considered and documented as such. An impaired level of consciousness should lead to the assessment of differential diagnoses of LM progression, for example, seizures or toxicity of medication. A complete examination of LM symptoms and signs can only be performed in patients without altered consciousness. Ideally, the same rater, or at least a limited number of raters, should perform the consecutive evaluations for 1 patient.

### Selection of the Domains to Assess

Contemporary data detailing the range of the clinical neurological status at the time of the diagnosis of LM and during the course of the disease are missing. The task force finally opted (by consensus) to select 14 domains for the new scorecard: level of consciousness, cognition, nausea and vomiting, vision, eye movement, facial strength, hearing, swallowing, speech, limb strength, limb ataxia, walking, bladder function, and bowel function (Table 1). Headache and pain, although not disputed as typical signs of LM, were felt to be highly variable and depend on several factors, such as symptomatic treatment (eg, CSF shunt) or extra-CNS disease, and cannot be easily measured. Therefore, these items were not included in the NANO-LM score. The following items were removed from the initial RANO-LM proposal: sensation and behavior. Sensation was associated with a moderate interobserver agreement in the effort of the validation of the NANO scale but was felt as a domain that has only low priority due to frequent treatment-induced neuropathies and limited objectivity potentially compromising a longitudinal assessment. Behavior, an output likely influenced by current psychological status, cognitive impairment, concomitant medications, and also the tumor itself had the least chance-adjusted interobserver concordance in the effort of the validation of the NANO scale. The language domain assessed in the NANO scale was considered as usually not related to LM and was thus not selected in the updated proposal. Gait, a domain in both the NANO scale and the first RANO-LM, was more clearly designated as walking. New domains felt of high interest in patients with LM were added: cognition, nausea and vomiting, ataxia (upper limb), and disturbances of bladder and bowel function.

After the identification of the domains, the task force discussed the best order to perform the assessment (Table 2). Importantly, the task force recommended the consideration of additional domains of interest as appropriate.

**Table 2.** NANO-LM clinical response assessment for leptomeningeal metastases

	
Complete response (CR)	Resolution of all symptoms and signs for all domains related to LM if the clinical evaluation was initially abnormal
Stable disease (SD)	All other situations not qualifying for CR or PD.
Progressive disease (PD)	Change from 0 to 2: in at least one domain or Change from 0 to 1 or from 1 to 2, in at least 3 domains

### Instructions for Each Domain

Instructions were provided for the assessment of each domain. Of note, the instructions provided for the assessment of the 6 domains shared by the NANO-LM and the RANO-LM task force are concordant (Supplementary Table 1). Some specifications were added to the items already present in the NANO scale and the first RANO-LM proposal (Table 1). Notably, instructions were provided for the assessment of level of consciousness and complementary definitions were provided for the evaluation of facial strength and limb strength.

For the assessment of limb strength, the rating for each limb should be noted on the scorecard. Similarly, vision, hearing, strength, or sensation of the face and finger-to-nose test should be tested and scored on both sides. The finger-to-nose test should be assessed only if the strength of the limb is not severely compromised (ie, strength 0 or 1). The assessment of vision should be performed while the patient is wearing corrective lenses or glasses, as applicable. Similarly, the assessment of hearing should be performed while the patient is wearing hearing devices, as applicable. The task force proposed to not document a swallowing evaluation, as proposed in the first RANO-LM effort. The group felt that a swallowing test with a small glass of water should not be performed because of the risk of acute complications (ie, aspiration).

### Rating of Each Domain

In the NANO scale, 9 domains are assessed and are rated between 0 and 2 for 4 domains and between 0 and 3 for 5 domains. In the initial RANO-LM effort, 11 domains are assessed and are rated between 0 and 2 for 4 domains and between 0 and 3 for 7 domains. The first RANO-LM proposal stated that transient deficits should be carefully assessed, as they may be primarily related to treatment. The task force proposes to regularly reassess fluctuating symptoms and signs until the resolution or development of a permanent deficit.

In the current NANO-LM proposal, the number of possible scores per item was limited to 3 options, 0 being normal and 2 being severe, to improve the reproducibility across raters in clinical trials. For each domain, any identified symptom or sign should be scored on the scorecard as “related to LM,” “possibly related to LM,” or “not related to LM.” The presumed condition other than LM causing the symptoms or signs should also be documented.

Neurological symptoms or signs should be described as they are and not according to the assumed anatomical origin. For example, lower limb paresis associated with urinary incontinence should not be reported as cauda equina syndrome but as 2 distinct signs; each cranial nerve deficit should be reported individually and not as cranial nerve impairment.

When a domain is rated as impaired during the follow-up, the rater should assess whether this observation is new, corresponds to significant progression of LM, is unchanged, or resolved. The task force proposed that neurological symptoms or signs that develop during the follow-up and that are related or suspected to be related to comorbidities or any other medical event should be described as nonevaluable.

If these signs and symptoms are retrospectively considered as LM-related and not to any comorbidity, the date of symptoms or signs appearance should be also retrospectively backdated to assess the response. In these situations, specific details should be included in the comments. Specific details on the clinical examination can additionally be included in the comments. If a domain evaluation was not performed, it should be scored as “not assessed.”

### Response Assessment

In the NANO scale, response assessment can be reported as neurologic response, neurologic stability, neurologic progression, not assessed, and nonevaluable.^17^ Corresponding definitions for NANO-LM are provided in Table 2. The NANO task force estimated that an overall score could overestimate tumor progression and tumor response and proposed that a significant change in any of the domains would be more appropriate to assess the response. Of note, the interobserver agreement for response assessment was not tested in the initial NANO effort.

In the initial RANO-LM proposal, progression was scored in the presence of a change of 2 or more levels in a single domain or by a change to the worse level (2 or 3) in any of the domains. The RANO-LM group acknowledged the need to consider potential differential diagnoses, notably related to brain metastases, extra-CNS disease, modification in comedications, or treatment-related toxicity.^13^

In the new NANO-LM proposal, the task force proposes that modifications in the prescription and use of steroids, analgesics, and antiemetics should be taken into account while performing the response assessment for each domain. During the follow-up, each symptom or sign should be rated as new onset, significant progression, unchanged, or resolved. A general overall assessment (improvement, stable, deterioration) should be done at the end of the clinical evaluation to compare the evolution since baseline or best clinical response.

Each domain has the same weight when performing the response assessment. A complete response is defined as a resolution of all signs and symptoms for all domains if the clinical evaluation was initially abnormal and attributed to LM. Progression corresponds to a change from 0 to 2 in any domain or a change from 0 to 1 or from 1 to 2 in at least 3 domains. Progression can be defined only if the neurological symptom or sign is related to LM. For example, a worsening from 0 to 2 for nausea in a patient who has reduced antiemetic medication should not be rated as progression. If worsening is considered as “possibly related to LM,” and then later confirmed as “related”: The date of progressive disease should be backdated. All other situations correspond to stable disease (Table 2). The task force felt that the category of “partial response” should be deleted to improve the reproducibility of the scorecard. Clinical responses are rare and therapeutic decisions rarely differ based on a difference between clinical partial response and clinical stable disease only.

## Discussion

Currently, no validated tool is available for the clinical neurological assessment of patients with LM. Here, the RANO-LM group proposes an updated scorecard for the clinical assessment of LM, the NANO-LM scale. The revised proposal considered the scales proposed by the NANO group^17^ for brain tumors and the first effort of the RANO-LM group to propose a clinical scorecard for LM.^13^ The current, updated NANO-LM scorecard needs validation. Indeed, a lack of reproducibility of the first MRI scorecard developed by the RANO-LM group^13^ has been shown^23^ and pitfalls were considered. Finally, the proposed updated EORTC-RANO-LM MRI scorecard was validated among 35 raters.^15^ The overall MRI-response assessment in LM patients perfectly matched the summary interpretation of the single ratings, as proposed in the scorecard instructions among 308 of 394 fully completed ratings. These results emphasized the need for clear instructions for scorecard use.

The interobserver concordance for the NANO scale for brain tumors was tested in 220 patients among 2 raters at 8 institutions.^17^ The interobserver agreement was measured by a kappa score ranging from −1 for no agreement to +1 for perfect agreement between observers, with 0 equating to agreement by chance and values below 0 showing an absence of agreement.^24,25^ The best kappa score showed an almost perfect agreement for language (kappa = 0.83), a substantial agreement for strength (kappa = 0.80) and gait (kappa = 0.76). The agreement was moderate for facial strength (kappa = 0.53), sensation (kappa = 0.50), ataxia (upper extremity) (kappa = 0.45), and fair for behavior (kappa = 0.35). Of note, the interobserver concordance rate for the level of consciousness could not be tested as only 1 patient had an altered level of consciousness documented.

The present report represents a first step and several challenges need to be considered and addressed. It is challenging to capture the complex clinical features of LM on a scorecard that is supposed to be useful for clinical practice. The clinical status assessment may have been oversimplified by allowing only 3 grades of being affected and patients with impaired consciousness would be difficult to assess. A prospective assessment for the NANO-LM scorecard will be important to evaluate the interobserver concordance for each item, the association between domains, and the interobserver concordance for the response assessment. The RANO-LM group proposes the validation of NANO-LM using an exploratory cohort and a validation cohort. The design and cohort sizes have been predefined, ethics approval has been obtained, and validation efforts are currently ongoing at several specialized centers (NCT06417710). Results from this study will inform on the value of the new scorecard for clinicians from various disciplines, both in clinical trials and in clinical practice for patients with LM, but broader applicability will still have to be explored.

## Supplementary material

Supplementary material is available online at *Neuro-Oncology* (https://academic.oup.com/neuro-oncology).

noae171_suppl_Supplementary_Tables_S1-S2
