# Supplementary material for: NANO-LM: An updated scorecard for the clinical assessment of patients with leptomeningeal metastases
Source: Neuro Oncol. 2024 Aug 29;27(2):455–65. doi: 10.1093/neuonc/noae171 (PMC11812256; doi:10.1093/neuonc/noae171)
Supplement: noae171_suppl_Supplementary_Tables_S1-S2 [file noae171_suppl_supplementary_tables_s1-s2.docx]

**NANO-LM: An updated scorecard for the clinical assessment of patients with leptomeningeal metastases**

**SUPPLEMENTARY MATERIAL**

Emilie Le Rhun, Lakshmi Nayak, Mary Jane Lim-Fat, Roberta Rudà, Elena Pentsova, Peter Forsyth, Barbara O'Brien, Matthias Preusser, Priya Kumthekar, Dieta Brandsma, Michael Weller

| Table S1 | Items from the NANO scale (Nayak et al., 2017) and from the RANO-LM group (Chamberlain et al., 2016) |
| --- | --- |
| Table S2 | Overall assessment of response in the NANO proposal (Nayak et al., 2017) and in the first RANO-LM proposal (Chamberlain et al., 2016) |

Table S1. Items from the NANO scale (Nayak et al. 2017) and from the initial RANO-LM group (Chamberlain et al. 2016)

| **NANO-LM** | |  | **RANO-LM** | |
| --- | --- | --- | --- | --- |
| **Domain, instructions** | **Scoring** |  | **Domain, instructions** | **Scoring** |
| **Gait**  Walking is ideally assessed by at least 10 steps | 0= normal  1= abnormal but walks without assistance  2= abnormal and requires assistance (companion, cane, walker, etc,)  3= unable to walk  🞎 not assessed  🞎 not evaluable |  | **Gait**  Walking is ideally assessed by at least 10 steps. | 0= normal  1= abnormal but walks without assistance  2= abnormal and requires assistance (companion, cane, walker, etc,)  3= unable to walk |
| **Strength**  Test each limb separately  Recommend assess proximal (above knee or elbow) and distal (below knee or elbow) major muscle groups  Score should reflect worst performing area Patients with baseline level 3 function in one major muscle group/limb can be scored based on  assessment of other major muscle groups/limb | 0= normal  1= movement present but decreased against resistance  2= movement present but none against resistance  3= no movement  🞎 not assessed  🞎 not evaluable |  | **Strength**  Each limb should be tested separately.  Recommend assess proximal (above knee or elbow) and distal (below knee or elbow) major muscle groups.  Score should reflect worst performing area.  Patients with preexisting level 3 function in one major muscle group/limb at baseline can be scored based on assessment of other major muscle groups/limb. | 0= normal  1= movement present but decreased against resistance  2= movement present but none against resistance  3= no movement |
| **Ataxia**  Non-evaluable if strength is compromised Trunk/lower extremities assessed by gait domain  Particularly important for patients with brainstem and cerebellar tumors  Score based on best response of at least 3 attempts | 0= able to finger to nose without difficulty  1= able to finger to nose but difficult  2= unable to finger to nose touch  🞎 not assessed  🞎 not evaluable |  |  |  |
| **Sensation**  Recommend evaluating major body areas separately  (face, limbs and trunk)  Score should reflect worst performing area  Sensory modality includes but not limited to light touch, pinprick, temperature and proprioception  Patients with baseline level 2 function in one major body area can be scored based on assessment of other major body areas | 0= normal  1= decreased but aware of sensory modality  2= unaware of sensory modality  🞎 not assessed  🞎 not evaluable |  | **Sensation**  Recommend evaluating major body  areas separately (face, limbs, and  trunk).  Score should reflect worst performing area.  Sensory modality includes but is not  limited to light touch, pinprick, temperature, and proprioception.  Patients with preexisting level 2  function in one major body area at  baseline can be scored based on  assessment of other major body  areas. | 0= normal  1= decreased but aware of sensory modality  2= unaware of sensory modality |
| **Visual fields**  Patients who requires correctives lenses should be evaluated while wearing corrective lenses  Each eye should be evaluated and score should reflect the worst performing eye | 0= normal  1= inconsistent or equivocal partial hemianopsia (> quadranopsia)  2= consistent or unequivocal partial hemianopsia (> quadranopsia)  3= complete hemianopsia  🞎 not assessed  🞎 not evaluable |  | **Vision**  Patients who require corrective lenses  should be evaluated while wearing  corrective lenses.  Each eye should be evaluated, and  score should reflect worst performing  eye. | 0= normal  1= partial monocular visual loss  2= complete monocular visual loss  3= bilateral visual loss |
|  |  |  | **Eye movements**  Test eye movements for each eye  individually.  The score will reflect the worst performing  eye (ie, the highest score). | 0= normal  1= abnormality noted in 1 direction of gaze  2= abnormality noted in more than 1 gaze direction, but not all  3= unable to move the eyes in any gaze direction |
| **Facial strength**  Particularly important for brainstem tumors  Weakness includes nasolabial fold flattening, asymmetric smile and difficulty elevating eyebrows | 0= normal  1= mild / moderate weakness  2= severe facial weakness  🞎 not assessed  🞎 not evaluable |  | **Facial strength**  Weakness includes nasolabial fold  flattening, asymmetric smile, and difficulty elevating eyebrow. | 0= normal  1= mild facial weakness (nasolabial fold flattening, asymmetric smile, decreased forehead contraction, or partial eye closure)  2= severe facial weakness (severe nasolabial fold flattening, asymmetric smile with limited or no movement of face, incomplete eye closure, or labial incompetence  3= bilateral facial  weakness |
| **Language**  Access based on spoken speech. Non-verbal cues or writing should not be included  Level 1: includes word finding difficulty; few paraphasic rrors/neologisms/word substitutions; but able to form sentences (full/broken)  Level 2: includes inability to form sentences (<4 words per phrase/sentence); limited word output: fluent but “empty” speech | 0= normal  1= abnormal but easily conveys meaning to examiner  2= abnormal and difficulty conveying meaning to examiner  3= abnormal. If verbal, unable to convey meaning to examiner OR non-verbal (mute/global aphasia)  🞎 not assessed  🞎 not evaluable |  |  |  |
|  |  |  | **Hearing**  Each ear should be evaluated and  score should reflect worst performing  ear. | 0= normal  1= impaired but residual serviceable  hearing  2= absent unilateral hearing  3= bilateral hearing loss |
|  |  |  | **Swallowing**  Bedside testing comprising a swallow  test with a small glass of water. | 0= normal  1= impaired but not requiring change  in diet formulation, not aspirating by  bedside testing  2= unable to swallow without risk of aspiration by bedside testing |
| **Level of consciousness**  None | 0= normal  1= drowsy (easily arousable)  2= somnolent (difficult to arouse)  3= unarousable/coma  🞎 not assessed  🞎 not evaluable |  | **Level of consciousness** | 0= normal  1= drowsy (easily arousable & responsive)  2= somnolent (difficult to arouse & poorly responsive)  3= coma (unrasouble & unresponsive) |
| **Behavior**  Particularly important for frontal lobe tumors  Alteration includes but is not limited to apathy, disinhibition and confusion  Consider subclinical seizure for significant alteration | 0= normal  1= mild/moderate alteration  2= severe alteration  🞎 not assessed  🞎 not evaluable |  | **Behavior**  Alteration includes but is not limited  to apathy, disinhibition, and confusion.  Consider subclinical seizures for  significant alteration. | 0= normal  1= mild/moderate alteration  2= severe alteration |
|  |  |  | Other | 0= normal  1= occasional or mild  2= severe alteration |

Table S2. Overall assessment of response in the NANO proposal (Nayak et al. 2017) and in the initial RANO-LM proposal (Chamberlain et al. 2016)

|  |  | NANO |  | First RANO-LM proposal |
| --- | --- | --- | --- | --- |
| Neurologic response |  | a ≥2 level improvement in at least one domain without worsening in other domains from baseline  or best level of function that is not attributable to change in concurrent medications or recovery from a comorbid event. |  | Improved; +2 to +3 in symptom inventory |
| Neurologic stability |  | a score of neurologic function that does not meet criteria for neurologic response, neurologic progression, non-evaluable, or not assessed. |  | no change (−1 to +1) in symptom inventory |
| Neurologic progression |  | a ≥2 level worsening from baseline or best level of function within ≥1 domain  or  worsening to the highest score within ≥1 domain that is felt to be related to underlying tumor progression and not attributable to a comorbid event or change in concurrent medication.  Of note, an assessment of neurologic progression does not require evaluation of a minimum number of domains of the NANO scale if any of these conditions is met. |  | a change of 2 or more levels in a given domain (eg, gait)  or alternatively by a change to level 3 (or level 2 in domains  defined by only 3 levels) in any one domain  −2 to −3 in symptom inventory |
| Non-evaluable |  | if it is more likely than not that a factor other than underlying tumor activity contributed to an observed change in neurologic function.  Such factors may include changes in a concurrent medication, such as corticosteroids, sedatives, narcotics, or anti-epileptic agents; acute or chronic adverse events related to therapeutic interventions; or a comorbid event such as a toxic-metabolic encephalopathy, post-ictal state, stroke, etc.  Non-evaluable could also be selected if measurement of a given domain is not feasible due to an alteration of another domain. For example, assessment of upper  extremity ataxia may not be possible if weakness of the extremity limits mobility. In this case, the strength domain should be assigned a numeric score but the upper extremity ataxia domain would be scored as non-evaluable. |  |  |
| Not assessed |  | if the clinician omits evaluation of that particular domain during his/her examination.  If a particular domain is marked not assessed at baseline, then that domain cannot thereafter be considered for progression or response. In general, assessment and scoring of all domains is encouraged. |  |  |

**References**

Chamberlain, Marc, Larry Junck, Dieta Brandsma, Riccardo Soffietti, Roberta Rudà, Jeffrey Raizer, Willem Boogerd, et al. 2016. “Leptomeningeal Metastases: A RANO Proposal for Response Criteria.” *Neuro-Oncology*, December. https://doi.org/10.1093/neuonc/now183.

Nayak, Lakshmi, Lisa M. DeAngelis, Alba A. Brandes, David M. Peereboom, Evanthia Galanis, Nancy U. Lin, Riccardo Soffietti, et al. 2017. “The Neurologic Assessment in Neuro-Oncology (NANO) Scale: A Tool to Assess Neurologic Function for Integration into the Response Assessment in Neuro-Oncology (RANO) Criteria.” *Neuro-Oncology* 19 (5): 625–35. https://doi.org/10.1093/neuonc/nox029.
